# FANCI plays an essential role in spermatogenesis and regulates meiotic histone methylation

**DOI:** 10.1038/s41419-021-04034-7

**Published:** 2021-08-09

**Authors:** Lan Xu, Weiwei Xu, Duan Li, Xiaoxia Yu, Fei Gao, Yingying Qin, Yajuan Yang, Shidou Zhao

**Affiliations:** 1 Center for Reproductive Medicine, Cheeloo College of Medicine, Shandong University, Jinan, Shandong 250012 China; 2 Key laboratory of Reproductive Endocrinology of Ministry of Education, Shandong University, Jinan, Shandong 250012 China; 3 Shandong Key Laboratory of Reproductive Medicine, Jinan, Shandong 250012 China; 4 Shandong Provincial Clinical Research Center for Reproductive Health, Jinan, Shandong 250012 China; 5 National Research Center for Assisted Reproductive Technology and Reproductive Genetics, Shandong University, Jinan, Shandong 250012 China; 6 State Key Laboratory of Stem Cell and Reproductive Biology, Institute of Zoology, Chinese Academy of Sciences, Beijing, 100101 China; 7 University of Chinese Academy of Sciences, Beijing, 100101 China; 8 State Key Laboratory of Genetic Resources and Evolution, Kunming Institute of Zoology, Chinese Academy of Sciences, Kunming, Yunnan, 650223 China; 9 Yunnan Key Laboratory of Animal Reproduction, Kunming Institute of Zoology, Chinese Academy of Sciences, Kunming, Yunnan, 650223 China

**Keywords:** Cell death, Spermatogenesis

## Abstract

FANCI is an essential component of Fanconi anemia pathway, which is responsible for the repair of DNA interstrand cross-links (ICLs). As an evolutionarily related partner of FANCD2, FANCI functions together with FANCD2 downstream of FA core complex. Currently, growing evidences showed that the essential role of FA pathway in male fertility. However, the underlying mechanisms for FANCI in regulating spermatogenesis remain unclear. In the present study, we found that the male *Fanci*^*−/−*^ mice were sterile and exhibited abnormal spermatogenesis, including massive germ cell apoptosis in seminiferous tubules and dramatically decreased number of sperms in epididymis. Besides, FANCI deletion impaired maintenance of undifferentiated spermatogonia. Further investigation indicated that FANCI was essential for FANCD2 foci formation and regulated H3K4 and H3K9 methylation on meiotic sex chromosomes. These findings elucidate the role and mechanism of FANCI during spermatogenesis in mice and provide new insights into the etiology and molecular basis of nonobstructive azoospermia.

## Introduction

Fanconi anemia (FA) is a rare genome instability syndrome characterized by early-onset bone marrow failure, developmental defects, high predisposition to cancers, and reduced fertility [1]. It has been revealed that mutations in FA genes, which are involved in DNA interstrand cross-links (ICLs) repair, usually lead to FA [2–4]. The FA pathway consists of 22 identified FA proteins (FANCA-FANCW) and several FA-associated proteins such as FAAP24 and FAAP100 [1, 5–8]. When DNA ICLs occur, FANCM recognizes the damage sites and recruits FA core complex proteins, which then monoubiquitinates FANCI-FANCD2 heterodimer [9, 10]. The monoubiquitination of the heterodimer is the keynote of FA pathway activation. Monoubiquitinated FANCI and FANCD2 then recruit downstream repair factors such as SLX4 (FANCP), XPF (FANCQ), BRCA1 (FANCS), BRCA2 (FANCD1), and RAD51 (FANCR) to complete the ICLs repair [11–15].

Currently, there are growing evidences for FA gene-associated infertility. It has been reported that mutations in FA genes including *FANCA* [16], *FANCM* [17], and *FANCU* [18] cause isolated non-obstructive azoospermia (NOA) in male patients. Consistently, many FA gene knockout mice showed sterility and hypogonadism in both males and females, and there were severe loss of germ cells and defects in spermatogenesis in male mice [19–23]. Besides, recent studies have reported that a network of FA proteins and DNA damage response (DDR) factors, MDC1 and RNF8, regulates the epigenetic modifications during meiosis, and FANCD2 is reported to be the central component of this network [24, 25]. Among FA proteins, FANCI is an evolutionarily related partner of FANCD2 and functions together with FANCD2 downstream of FA core complex to repair ICLs [26]. However, the exact role and mechanisms of FANCI, which is critical for FA pathway activation, in male meiosis remain unclear.

In the present study, we generated *Fanci*^−/−^ mice and found that the male *Fanci*^−/−^ mice were sterile and exhibited aberrant spermatogenesis. Further studies indicated that FANCI was essential for FANCD2 foci formulation and regulated histone methylation during meiosis. Altogether, these findings show that FANCI, an important component of FA pathway, plays an essential role in spermatogenesis.

## Materials and methods

### Animals

*Fanci-flag* mice with 3×flag fusing to N-terminus of the FANCI protein were generated using the CRISPR/Cas9 technology. The detailed information of the generation of the *Fanci-flag* knock-in mouse was shown in Supplementary Fig. 1. *Fanci*^*−/−*^ mice were generated by Nanjing Sync Biotech Company using the CRISPR/Cas9 technology. The deletion of 98 bp in exon 5 resulted in frameshift mutation of *Fanci* gene, which led to a truncated protein of 116 amino acids (Supplementary Fig. 2). Primer sequences were as follows: *Fanci-flag*, forward: TCTGTTGAATGGATGGTGAAGATGT, reverse: TGGGGCATGTGTACACCGAG; *Fanci* knockout, forward: CCTAACCTTTGAAGCCCCTCG, reverse: GGGTGTCAATCAACTGCCTC.

### Histology, immunohistochemistry, and TUNEL staining

Testes from control and *Fanci*^*−/−*^ mice were fixed in 4% PFA overnight, and then dehydrated and embedded in paraffin. For morphological analysis, paraffin sections at 5 μm thick were stained with H&E. For immunohistochemistry, sections were incubated at 4 °C overnight with primary antibodies, after that the sections were incubated with specific secondary antibodies (Zhongshan Biotech) and then stained with DAB Substrate Kit (Vectorlabs). For the TUNEL assay, sections were treated with 15 μg/ml proteinase K for 10 min at 37 °C and then stained with the in situ Cell Death Detection kit (Roche). Images of histological analysis were captured on Olympus BX53 fluorescence microscope.

### Meiotic chromosome spread and immunofluorescence microscopy

Meiotic chromosome spread of spermatocytes was performed as previously described [27]. Briefly, seminiferous tubules were incubated in hypotonic buffer (30 mM Tris (pH 7.5), 50 mM sucrose, 17 mM trisodium citrate, 5 mM EDTA, 1 mM PMSF, and 2.5 mM dithiothreitol) for 30 min and then minced in 100 mM sucrose to release spermatocytes. After that, the suspension was spread on slides with 1% PFA and 0.1% Triton X-100. Slides were then incubated in a humid chamber at 4 °C overnight. The following antibodies were used: SOX9 (Sigma, 1:500), DDX4 (Abcam, 1:500), PLZF (Santa Cruz, 1:100), FLAG (Sigma 1:100), FANCD2 (Novus, 1:200), SYCP1 (Abcam, 1:500), SYCP3 (Abcam, 1:500), γH2AX (Cell Signaling Technology, 1:200), RNA Pol II (Sigma, 1:200), RAD51 (Invitrogen, 1:200), MLH1 (BD Biosciences, 1:100), H3K9me2 (Millipore, 1:500), H3K9me3 (Millipore, 1:500), H3K4me2 (Millipore, 1:200) and cleaved PARP1 (Cell Signaling Technology, 1:400). FITC- and TRITC-conjugated secondary antibodies (Invitrogen) were used. Fluorescence images were captured with the Dragonfly spinning disc confocal microscope (ANDOR Technology).

Meiotic stages were determined on the basis of SYCP3 signal as previously described [24]. Briefly, spermatocytes in leptotene have short and unsynapsed SYCP3 signal. In zygotene spermatocytes, homologous chromosomes begin to pair, and the SYCP3 signal on lateral elements becomes longer. At pachytene, homologous chromosomes fully synapse, and the SYCP3 signal is thick and condensed. In the diplotene stage, the SYCP3 signal is compact on XY chromosomes and decreases progressively on autosomes because of the desynapse of homologous chromosomes.

The relative mean fluorescence intensity (RMFI) of histone methylation was quantified using the ImageJ software. Briefly, regions of interest (ROIs) were drawn around XY chromatin, presented as XY in Fig. 8, and the other nuclear area excluding the XY body was presented as Autosome (AU) in Fig. 8. The ROIs of XY chromatin and autosome region were standardized to the background. In this study, we calculated the RMFI of spermatocytes in both pachytene and diplotene stages.

### Protein extraction and western blotting

Total protein of testes was extracted with the Total Protein Extraction Kit (Invent) and the protein concentration was examined by BCA method (Thermo). Protein samples were separated by SDS-PAGE and then transferred to PVDF membrane (Millipore). After blocking in 5% milk, the membranes were incubated with indicated primary antibodies at 4 °C overnight. The following antibodies were used: FANCI (Novus, 1:5000), FLAG (Sigma, 1:5000), and α-Tubulin (Proteintech, 1:3000). After being incubated with secondary antibodies, the membranes were detected with an ECL system (Millipore). The ChemiDoc MP System (Bio-Rad) was used to capture images.

### Quantitative RT-PCR analysis

Total RNA from tissues was extracted with Trizol reagent (Thermo) and then reverse-transcribed using a PrimeScript RT Reagent Kit (TAKARA). The mRNA level of *Fanci* was quantitated by real-time PCR using the LightCycler 480 SYBR Green I Master Mix (Roche). *Gapdh* was used as the internal control. Primer sequences were as follows: *Fanci*, forward: CATCTTGATGGATTCCTATGGGC, reverse: GTTCGCGTAACAACTCTGTTGA; *Gapdh*, forward: AATGGATTTGGACGCATTGGT, reverse: TTTGCACTGGTACGTGTTGAT. Independent experiments were conducted three times.

### mRNA probe analysis

Paraffin testicular sections were pretreated and analyzed with *Fanci* BaseScope™ Probe (ACD), which was a method of in situ hybridization (ISH) to visualize mRNA. The RNA-specific probe was designed to detect the 98 bp which was deleted in *Fanci*^*−*^^*/−*^ mice. According to the manufacturer’s instructions, testicular sections were first pretreated with reagents such as hydrogen peroxide and protease, and then incubated with RNA-specific probe. After that, the sections were incubated with amplification reagents to enhance signals, and the signals were further detected using a red chromogenic substrate. Images were acquired with the Olympus BX53 microscope.

### Statistical analysis

Data in this study were analyzed using the SPSS version 21.0. Numeric variables were compared by Student’s *t*-test. For categorical variables, the Chi-square test (Fisher’s exact test) was used to compare the differences. *P* < 0.05 was considered statistically significant.

## Results

### Expression pattern of FANCI during meiotic prophase I

**Fig. 1:**
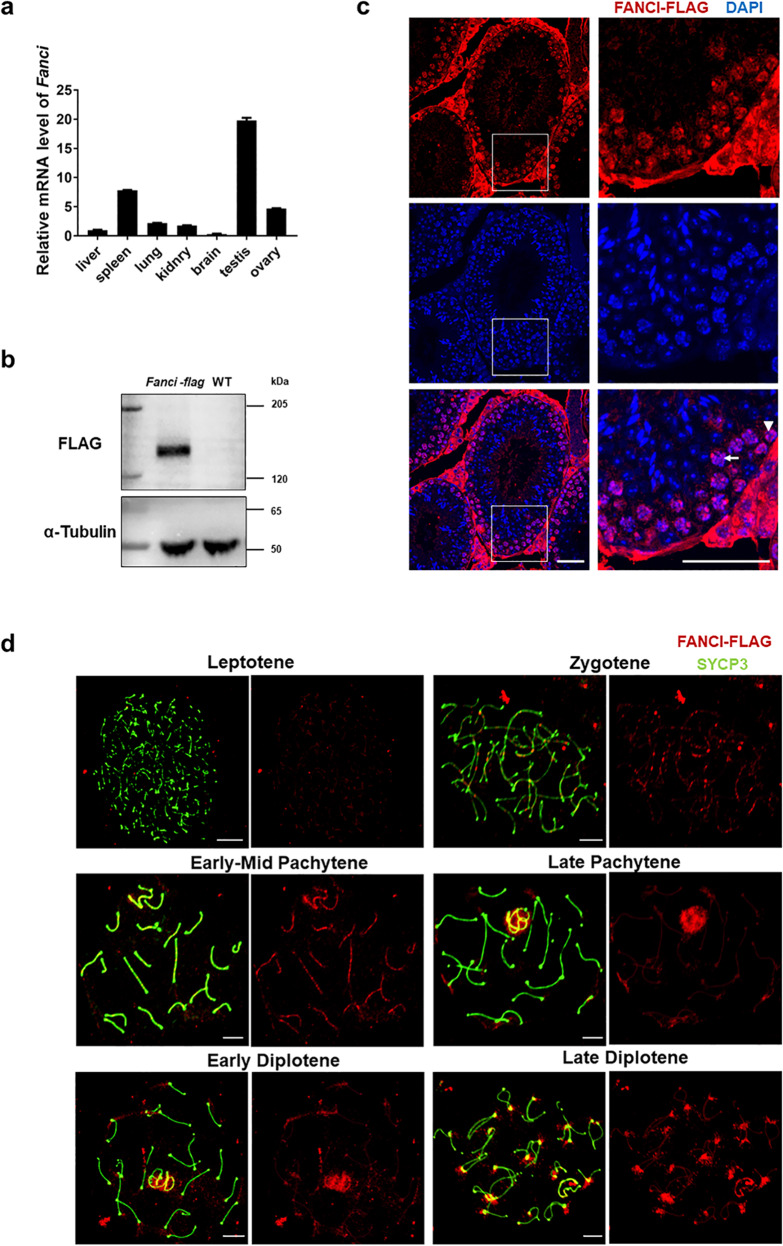
Temporal and spatial expression of FANCI during meiotic prophase I. **a** Relative mRNA level of *Fanci* in various tissues. Data are presented as mean ± SD. **b** Western blot analysis of the wild type and *Fanci-flag* testes (8 weeks old). α-Tubulin was used as a loading control. **c** Expression and localization of FANCI in adult testes. Immunofluorescence analysis of FLAG was performed on paraffin sections from *Fanci-flag* testes. The nuclei were stained with DAPI. Arrowhead represents spermatogonia. Arrow represents spermatocyte. Scale bars, 20 μm. **d** Localization of FANCI in chromosome spread of *Fanci-flag* spermatocytes. Meiotic chromosome spreads were immunolabeled with anti-FLAG and anti-SYCP3 antibodies. Scale bars, 5 μm.

Measurement of mRNA levels by real-time PCR showed that *Fanci* was differentially expressed in various mouse tissues, with much higher level in testis than other tissues (Fig. 1a).

We next detected the localization and expression of FANCI by generating a *Fanci-Flag* mouse model (Fig. 1b, Supplementary Fig. 1). The *Fanci-Flag* mice were normal in appearance. The body weight and the testis/body ratio of adult mice were comparable between wild-type and *Fanci-Flag* mice. Besides, both *Fanci-Flag* males and females were fertile (Supplementary Fig. 1d−g). Immunofluorescence staining of testicular sections from *Fanci-Flag* mice showed that FANCI was localized in the nuclei of spermatogonia and spermatocytes (Fig. 1c). Consistent with our results, a single-cell RNA-seq analysis also indicated that *Fanci* had relatively high expression levels in spermatogonia and spermatocyte (Supplementary Fig. 3) [28]. Immunofluorescence microscopy of meiotic chromosome spreads from *Fanci-Flag* mice was performed and each meiotic stage was determined on the appearance pattern of chromosome axes. Results showed that FANCI was not detected in the leptotene stage. Its accumulation on the chromosome axes began in the zygotene stage and increased through the mid-pachytene stage. After mid-pachytene stage, FANCI signal on the autosome region gradually decreased. Meanwhile, FANCI signal on the sex chromosomes progressively increased and spread onto the entire XY domain in the early diplotene stage. After that FANCI signal decreased on the XY domain and diffused throughout the entire chromatin in the late diplotene stage (Fig. 1c). These results indicated that FANCI was expressed in a temporal and spatial manner during meiotic prophase I.

### FANCI deletion results in male sterility and causes germ cell loss in mice

**Fig. 2:**
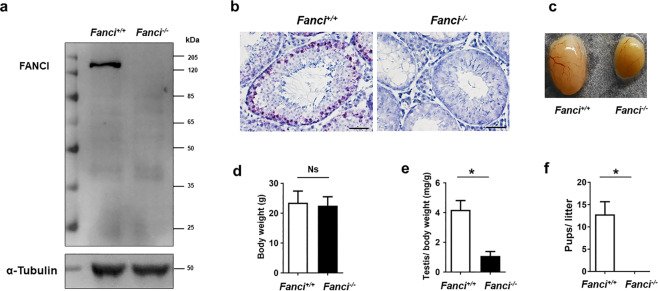
FANCI deletion results in atrophytestes and male sterility. **a** Western blot analysis of the wild type and *Fanci*^*−/−*^ testes (8 weeks old). α-Tubulin was used as a loading control. **b** The mRNA probe detection of wild type and *Fanci*^*−/−*^ testes. Scale bars, 20 μm. **c** Representative images of the testes of wild-type and *Fanci*^*−/−*^ mice. **d** Body weights of wild-type and *Fanci*^*−/−*^ mice at 8 weeks after birth. Six mice were analyzed for each group, and data are presented as mean ± SD. *, *P* < 0.05. Student’s *t*-test. **e** Mean ratio of testis/body weight from 8 weeks old wild type and *Fanci*^*−/−*^ mice. Six mice were analyzed for each group, and data are presented as mean ± SD. *, *P* < 0.05. Student’s *t*-test. **f** Fertility test result of the mean ratio of pups/litter from wild type and *Fanci*^*−/−*^ mice. Six wild-type mice: 34 litters and 12.8 pups/litter. Six *Fanci*^*−/−*^ mice: 0 litter. Data are presented as mean ± SD. *, *P* < 0.05. Student’s *t*-test.

To explore the function of FANCI in male fertility, we generated *Fanci*^*−/−*^ mice by targeted deletion of 98 bp in exon 5 using the CRISPR/Cas9 technology (Supplementary Fig. S2). FANCI was not detected by western blotting in *Fanci*^*−/−*^ testes (Fig. 2a). Furthermore, the results of mRNA probe did not show any signal in the testis of *Fanci*^*−/−*^ mice (Fig. 2b).

Genotyping of offspring from heterozygous mating showed a normal Mendelian distribution (Supplementary Fig. 2d). Besides, *Fanci*^*−/−*^ mice appeared to be normal without microphthalmia or skeletal malformations (Supplementary Fig. 2e−g). The body weight of adult mice was comparable between wild-type and *Fanci*^*−*^^*/−*^ mice (Fig. 2d). However, the *Fanci*^*−/−*^ testes were smaller than those of littermate controls, with the ratio of the testis to body weight decreased significantly (Fig. 2c, e). Furthermore, both *Fanci*^*−/−*^ males (Fig. 2f) and females were infertile.

**Fig. 3:**
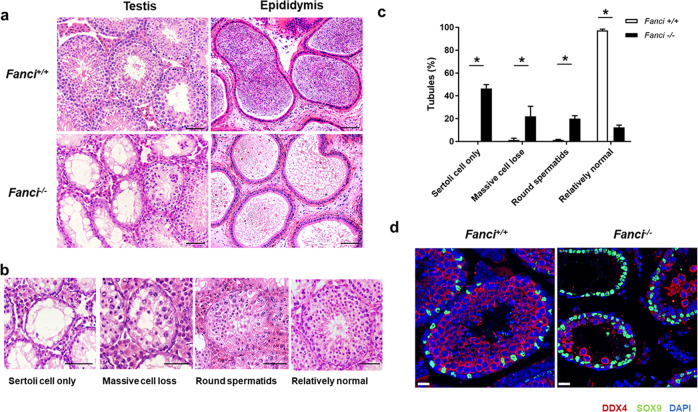
FANCI deletion causes germ cell loss in mice. **a** H&E staining of testes and epididymides from 8 weeks old wild type and *Fanci*^*−/−*^ mice. Scale bars, 50 μm. **b** Representative images of H&E stained testicular sections showing various seminiferous tubules in 8 weeks old *Fanci*^*−/−*^ mice, including Sertoli cell-only tubules, tubules with massive cell loss, tubules with round spermatids as the most advanced spermatogenic cells, and relatively normal tubules. Scale bars, 50 μm. **c** Quantification of different types of seminiferous tubules in 8 weeks old wild type and *Fanci*^*−*^^*/−*^ mice. Six wild-type mice and six *Fanci*^*−/−*^ mice were analyzed. Data are presented as mean ± SD. *, *P* < 0.05. Chi-square test (Fisher’s exact test). **d** Immunofluorescence staining for DDX4 (a germ cell marker) and SOX9 (a Sertoli cell marker) in wild type and *Fanci*^*−/−*^ mice testes of 8-week-old mice. Scale bars, 20 μm.

To further explore the functions of FANCI, we compared testis sections from 8 weeks old wild-type and *Fanci*^*−/−*^ mice using H&E staining. Seminiferous tubules from wild-type mice had normal architecture and contained germ cells at all stages. In contrast, seminiferous tubules from *Fanci*^*−/−*^ testes were predominantly degenerated and exhibited a significant decrease in the number of cells. Consistent with the histologic analysis of testis sections, sperms in epididymides of *Fanci*^*−/−*^ mice were much fewer than controls (Fig. 3a). Additionally, in *Fanci*^*−/−*^ testes, 46.3% of seminiferous tubules only contained Sertoli cells, and 22% of seminiferous tubules had massive cell loss. Moreover, 20% of *Fanci*^*−/−*^ tubules had round spermatids as the most advanced spermatogenic cells, and only 12.3% tubules in *Fanci*^*−/−*^ testes had relatively normal morphology (Fig. 3b, c). Furthermore, immunofluorescence staining of testicular sections for DDX4 (a germ cells marker) and SOX9 (a marker for Sertoli cells) indicated massive germ cell loss in *Fanci*^*−/−*^ testes (Fig. 3d), suggesting that FANCI deletion resulted in the dramatically decrease of germ cells and relative enrichment of Sertoli cells. In addition, massive loss of germ cells was found in *Fanci*^*−/−*^ testes at 12 months, which was more severe than that at 8 weeks (Supplementary Fig. 4a, c). Exactly, 60.7% of seminiferous tubules only contained Sertoli cells, and only 9.0% tubules had relatively normal morphology (Supplementary Fig. 4b). Collectively, our results indicate that FANCI is required for male fertility.

### FANCI deletion increases apoptosis of germ cells and affects the maintenance of undifferentiated spermatogonia

**Fig. 4:**
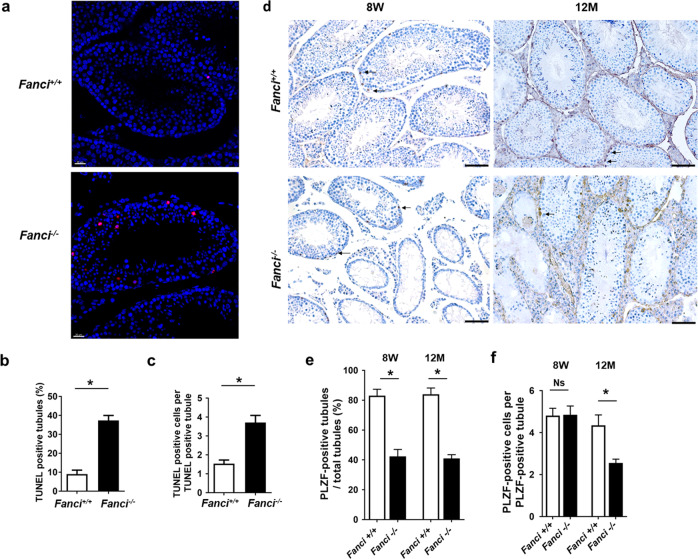
FANCI deletion increases apoptosis of germ cells and affects the maintenance of undifferentiated spermatogonia. **a** TUNEL assay of testes from 8-week-old wild-type and *Fanci*^*−/−*^ mice. Cells stained red are TUNEL-positive. Scale bars, 20 μm. **b** Frequencies of TUNEL-positive tubules in wild type and *Fanci*^*−/−*^ mice. In each group, six mice were analyzed. Data are presented as mean ± SD. *, Chi-square test (Fisher’s exact test). **c** Numbers of TUNEL-positive cells per TUNEL-positive tubule in wild type and *Fanci*^*−/−*^ mice. Six wild-type mice and six *Fanci*^*−*^^*/−*^ mice were analyzed. Data are presented as mean ± SD. *, *P* < 0.05. Student’s *t*-test. **d** Immunohistochemistry staining against PLZF (an undifferentiated spermatogonia marker) on wild type and *Fanci*^*−/−*^ testes at 8 weeks old and at 12 months old. Arrows represent the undifferentiated spermatogonia. Scale bars, 50 μm. Scoring of the percentage of PLZF-positive tubules per total tubule (**e**) and scoring of the number of PLZF-positive cells per PLZF-positive tubule (**f**). A total of 100 tubules from three independent mice were examined at 8 weeks and 12 months, both for wild type and *Fanci*^*−/−*^ mice. In each group, six mice were analyzed. Data are presented as mean ± SD. *, *P* < 0.05. Ns no statistical significant difference. Frequencies were compared by the Chi-square test (Fisher’s exact test). Numbers were compared by Student’s *t*-test.

Consistent with massive germ cell loss during spermatogenesis, TUNEL assay and immunofluorescence staining for cleaved PARP1 showed that apoptosis was dramatically increased in *Fanci*^*−/−*^ testes (Fig. 4a−c and Supplementary Fig. 5a−c), suggesting that *Fanci*^*−/−*^ germ cells were eliminated by increased apoptosis. Furthermore, we found that most cleaved PARP1 signals were localized in SYCP3-positive cells in *Fanci*^*−/−*^ testes, indicating that most apoptotic cells were spermatocytes. Only a few apoptotic cells were undifferentiated spermatogonia or spermatids in *Fanci*^*−/−*^ testes (Supplementary Fig. 5d, e).

As the maintenance of undifferentiated spermatogonia is required for continuous spermatogenesis, we next investigated whether the number of undifferentiated spermatogonia was altered in *Fanci*^*−/−*^ testes. Immunohistochemistry staining utilizing PLZF (an undifferentiated spermatogonia marker) showed that percentage of PLZF-positive tubules were significantly decreased in *Fanci*^*−/−*^ mice compared with controls at both 8 weeks and 12 months (Fig. 4d, e). Despite the number of PLZF-positive cells per PLZF-positive tubules was similar between *Fanci*^*−/−*^ and wild-type mice at 8 weeks, the average number of PLZF-positive cells decreased dramatically in *Fanci*^*−/−*^ testes at 12 months compared with wild type (Fig. 4d, f). These results suggest that FANCI plays an important role in the maintenance of undifferentiated spermatogonia.

### FANCI colocalizes with FANCD2 on sex chromosomes and is required for FANCD2 foci formation in spermatocytes

**Fig. 5:**
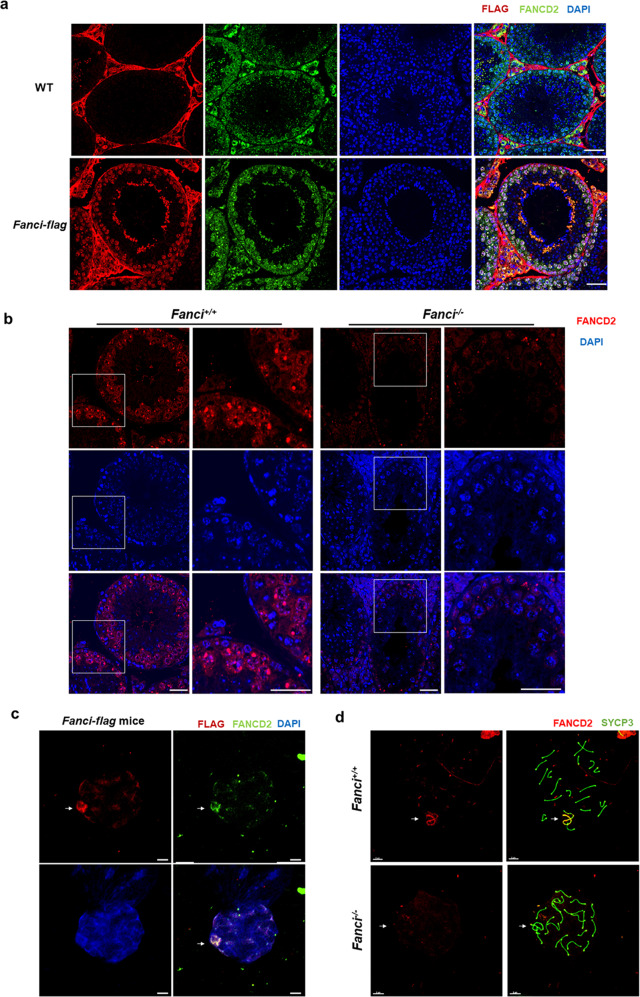
FANCI colocalizes with FANCD2 on sex chromosomes and is required for FANCD2 foci formation in spermatocytes. **a** Expression and localization of FANCI and FANCD2 in adult testes. Immunofluorescence analysis of FLAG and FANCD2 was performed on paraffin sections from wild type and *Fanci-flag* testes. The nuclei were stained with DAPI. Scale bars, 20 μm. **b** Immunostaining of paraffin sections from wild-type testes and *Fanci*^*−/−*^ testes with FANCD2. DNA was stained with DAPI. Scale bars, 20 μm. **c** Expression and localization of FANCI and FANCD2 in spermatocytes. Immunofluorescence analysis of FLAG and FANCD2 was performed on chromosome spread from *Fanci-flag* testes. The nuclei were stained with DAPI. Arrows represent the sex chromosomes. Scale bars, 5 μm. **d** Immunostaining of chromosome spreads from wild type and *Fanci*^*−/−*^ spermatocytes with FANCD2 and SYCP3. SYCP3 immunolabeling was used to distinguish meiotic stages. Scale bars, 5 μm.

It is well-known that FANCI and FANCD2 form a heterodimer and play a critical role in FA pathway activation. Thus, we firstly investigated the localization of FANCD2 and FANCI in *Fanci-Flag* mice, and found that FANCD2 and FANCI were mainly colocalized in spermatogonia and spermatocytes (Fig. 5a). Besides, nuclear spread analysis of spermatocytes from *Fanci-Flag* mice indicated that FANCD2 and FANCI were colocalized on sex chromosomes (Fig. 5c).

Next, we examined the expression of FANCD2 in wild type and *Fanci*^*−/−*^ testes. Results showed that FANCD2 expression was dramatically decreased in *Fanci*^*−/−*^ testes compared with wild-type testes, and the FANCD2 signal was attenuated in both spermatogonia and meiotic cells (Fig. 5b). Furthermore, immunofluorescence microscopy of meiotic chromosome spreads revealed that FANCD2 foci was eliminated in *Fanci*^*−/−*^ cells (Fig. 5d), suggesting that FANCI is required for FANCD2 foci formation in spermatocytes.

### Meiotic prophase I stage progression and meiotic recombination remain unaffected in *Fanci*^*−/−*^ spermatocytes

**Fig. 6:**
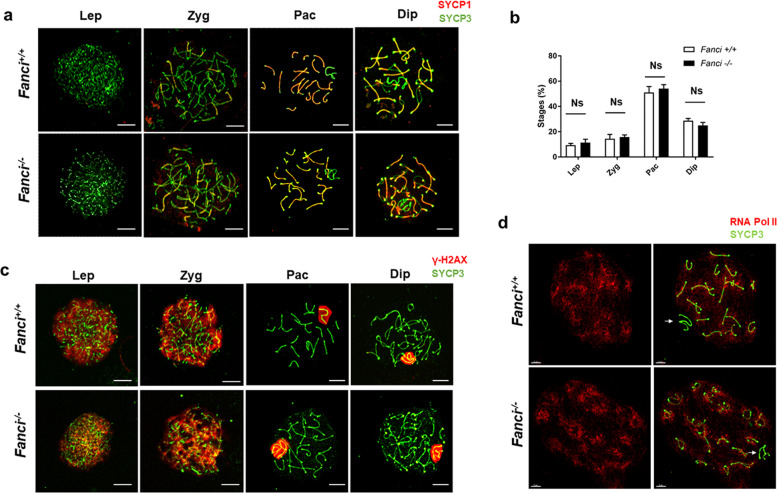
Meiotic prophase I stage progression and synapsis in *Fanci*^*−/−*^ spermatocytes. **a** Wild type and *Fanci*^*−/−*^ spermatocytes were immunolabeled with SYCP1 and SYCP3 in leptotene (Lep), zygotene (Zyg), pachytene (Pac), and diplotene (Dip) spermatocytes. Meiotic stages were determined on the basis of SYCP3 signals. **b** Percentage of each stage of meiotic prophase as determined by SYCP3 immunostaining. In each group, six mice were analyzed. Data are presented as mean ± SD. Ns no statistical significant difference. Chi-square test (Fisher’s exact test). **c** Wild type and *Fanci*^*−/−*^ spermatocytes were immunolabeled with γ-H2AX and SYCP3 at leptotene (Lep), zygotene (Zyg), pachytene (Pac), and diplotene (Dip) spermatocytes. **d** Spermatocytes immunolabeled for RNA Polymerase II (RNA Pol II) and SYCP3 at diplotene spermatocytes. Arrows represent the sex chromosomes. Scale bars, 5 μm.

**Fig. 7:**
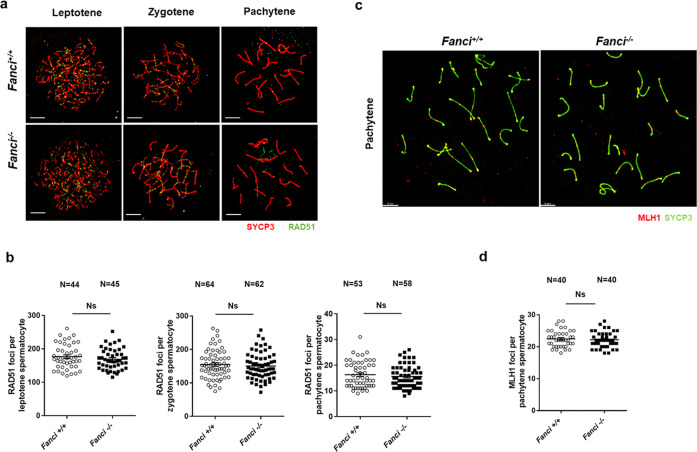
Meiotic recombination and crossover in *Fanci*^*−/−*^ spermatocytes. **a** Spermatocytes immunolabeled for RAD51 at leptotene, zygotene, and pachytene spermatocytes. **b** Quantification of the number of RAD51 foci per cell at leptotene, zygotene, and pachytene in wild type and *Fanci*^*−/−*^ spermatocytes. Total numbers of analyzed spermatocytes are indicated. Data are presented as mean ± SD. Ns no statistical significant difference. Student’s *t*-test. **c** Spermatocytes immunolabeled for MLH1 in Wild type and *Fanci*^*−/−*^ spermatocytes. **d** Quantification of the number of MLH1 foci per cell in wild type and *Fanci*^*−/−*^ spermatocytes. Total numbers of analyzed spermatocytes are indicated. Data are presented as mean ± SD. Ns no statistical significant difference. Student’s *t*-test.

To investigate the roles of FANCI in meiotic prophase I, we analyzed multiple meiosis-related markers in wild type and *Fanci*^*−/−*^ spermatocytes by immunofluorescence staining on meiotic chromosome spreads. As shown in Fig. 6a,b, normal chromosome synapsis and meiotic progression were observed in *Fanci*^*−/−*^ spermatocytes. Besides, a normal phosphorylated H2AX (γH2AX) signal was detected in all stages of meiotic prophase I in both wild-type and *Fanci*^*−/−*^ mice (Fig. 6c). To determine whether meiotic recombination was disturbed by FANCI deletion, we examined the accumulation of RAD51 on chromosome axes in *Fanci*^*−/−*^ spermatocytes. Results showed that the number of RAD51 foci were comparable between wild type and *Fanci*^*−/−*^ spermatocytes at both early stages (leptotene and zygotene) and later stage (pachytene) (Fig. 7a, b). Furthermore, we examined the accumulation of MLH1, which is the marker for meiotic crossover. We found that the number of MLH1 foci on chromosome axes were also comparable in both groups in pachytene spermatocytes (Fig. 7c, d), suggesting that crossover formation was unaffected in *Fanci*^*−/−*^ spermatocytes. Collectively, these results reflect that FANCI is dispensable for meiotic progression and meiotic recombination.

### FANCI regulates H3K4 and H3K9 methylation on meiotic sex chromosomes

**Fig. 8:**
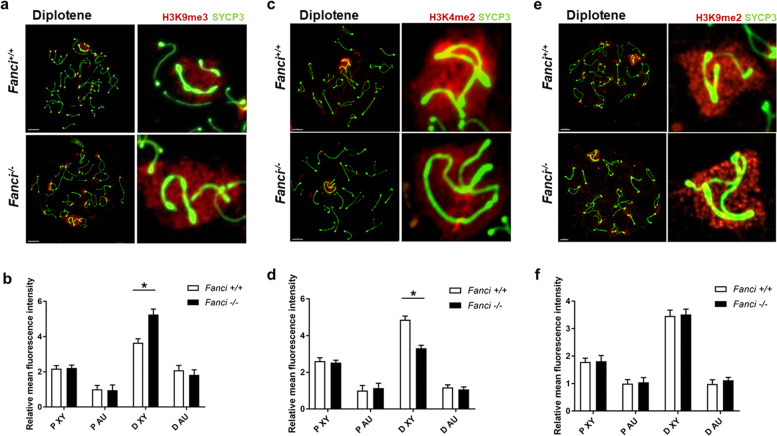
FANCI regulates epigenetic modification on sex chromosomes during meiosis. **a, c, e** Immunofluorescence analysis of meiotic chromosome spreads from wild type and *Fanci*^*-/-*^ mice with indicated antibodies. Areas surrounding sex chromosomes are magnified. Scale bars, 5 μm. Quantification of H3K9me3 (**b**), H3K4me2 (**d**), and H3K9me2 (**f**) relative mean fluorescence intensity (RMFI) on autosome regions (Au) and sex chromosomes (XY) in pachytene (P) and diplotene (D) spermatocytes. For each panel, 30 cells were analyzed for each group. Data are presented as mean ± SD. *, *P* < 0.05. Student’s *t*-test.

It has been reported that FA core proteins and FANCD2 regulate H3K4 and H3K9 methylation on meiotic sex chromosomes [24, 25]. To determine whether FANCI is involved in such histone methylation, we performed immunostaining for H3K4me2, H3K9me2, and H3K9me3 on chromosome spreads from wild type and *Fanci*^*−/−*^ spermatocytes and then quantified the RMFI both on the autosomes regions and XY body. Consistent with previous studies, the accumulation of H3K9me3 on sex chromosomes was increased in *Fanci*^*−/−*^ spermatocytes in the diplotene stage (Fig. 8a), indicating that FANCI was also involved in the regulation of H3K9 methylation. Besides, in *Fanci*^*−/−*^ spermatocytes, H3K4me2 intensity was decreased significantly on diplotene sex chromosomes (Fig. 8c). Furthermore, H3K9me2 on XY chromatin was unaffected during the pachytene-to-diplotene transition in *Fanci*^*−/*−^ spermatocytes (Fig. 8e). These results suggest that FANCI negatively regulates H3K9me3 and positively regulates H3K4me2 in the diplotene stage. To determine whether the changes in histone modifications in *Fanci*^*−/−*^ sex chromosomes are associated with transcriptional changes, we examined the distribution of RNA polymerase II (RNA Pol II) in diplotene spermatocytes. Surprisingly, RNA Pol II signal was excluded from the sex body in diplotene spermatocytes in both wild type and *Fanci*^*−/−*^ mice (Fig. 6d), suggesting a normal sex chromosome transcriptional inactivation in *Fanci*^*−/−*^ spermatocytes.

## Discussion

In this study, we elucidated temporal and spatial expression of FANCI during meiotic prophase I. Besides, FANCI deletion eliminated FANCD2 foci and altered histone methylation during meiosis, which contributed to the spermatogenetic failure in *Fanci*^*−/−*^ mice. Collectively, our results elucidate the involvement and mechanism of FANCI during spermatogenesis.

During spermatogenesis, FANCI was reported to localize in spermatocytes and spermatids [29]. However, the exact expression patterns and localization of FANCI during meiotic prophase I are unclear. In the present study, we revealed the exact expression patterns and localization of FANCI from leptotene to late diplotene for the first time. We found that FANCI localized on the chromosome axes from zygotene to mid pachytene stage, after that FANCI signal accumulated on the XY region from late pachytene to early diplotene stage, and then it diffused throughout the entire chromatin in the late diplotene stage. These findings indicated the temporal and spatial manner of FANCI expression and may provide information for future functional studies.

Importantly, we demonstrated that *Fanci*^*−/−*^ mice exhibited germline defects including massive germ cell loss and increased apoptosis in seminiferous tubules. The germline phenotypes and compromised fertility in *Fanci*^*−/−*^ mice in our study are consistent with many previous FA mouse models [22, 23, 25], indicating that FANCI, as an important component of FA pathway, also plays essential roles in the male germline. Previous studies have revealed that FA proteins were involved in the maintenance of hematopoietic stem cells and human pluripotent stem cells [30, 31], and it is reasonable that FA proteins are associated with undifferentiated spermatogonia. Kato et al. [25] indicated that FANCB regulated maintenance of undifferentiated spermatogonia. Consistent with this study, our findings also suggest an important role for FANCI in the maintenance of undifferentiated spermatogonia, but the underlying mechanism is uncertain and requires further exploration.

It is well-known that FA pathway is a DDR pathway that repairs DNA ICLs in the genome [1]. During meiosis, one important DDR event is meiotic recombination [32]. However, our results demonstrated that FANCI deletion did not affect the number of RAD51 foci on chromosome axes from leptotene to pachytene, suggesting an unessential role of FANCI in RAD51 recruitment and exclusion during meiosis. Similar results were also observed in *Fancb* mutant mice [25]. Despite the unessential role of FANCI in RAD51 accumulation during meiosis, many studies have revealed that FANCI is involved in the regulation of RAD51 functions during DDR. Studies indicated that FANCI-FANCD2 complex colocalized with RAD51 at the stalled replication forks and stabilizes the RAD51-DNA complex [33–35]. Besides, Sato et al. indicated that FANCI, but not FANCD2, was essential for the FANCI-FANCD2 complex-mediated RAD51-DNA stabilization [36]. Furthermore, a recent study also claimed that FANCI interacted directly with RAD51 and stimulated RAD51-mediated D-loop formation [29]. The underlying mechanism behind this difference is unclear and requires further exploration. Additionally, we also found the normal γ-H2AX and MLH1 signal in *Fanci*^*−/−*^ spermatocytes from leptotene to pachytene, which is consistent with the observation in *Fancb* mutant mice [25]. Collectively, our results revealed the normal programmed DSB repair and meiotic recombination in *Fanci*^*−/−*^ mice, suggesting a dispensable role of FANCI in these events.

Among the FA proteins, FANCI and FANCD2 form a protein complex (ID2 complex), and the monoubiquitination of ID2 complex is a critical step in FA pathway activation during ICLs repair [37]. When it comes to spermatogenesis, our results indicated that the global FANCD2 level was decreased in *Fanci*^*−/−*^ testes. One possible reason for the global decreased FANCD2 is that FANCI and FANCD2 are partially interdependent for their protein stability [38]. In line with our findings, a previous study has also reported that meiotic FANCD2 chromosomal localization was dependent on FANCI in *C. elegans*, suggesting the important role of FANCI during meiosis in different species [39]. When it comes to spermatogenesis, recent studies reported that FANCD2 foci on chromosome axes were abolished in mutant spermatocytes deficient for FANCA, FANCB, and FANCC [24, 25], indicating the similar function of FA core complex in regulating FANCD2 foci in both somatic DNA damage response DDR and meiosis. In line with these studies, our results showed that FANCD2 foci were eliminated in *Fanci*^*−/−*^ spermatocytes, indicating that FA pathway activation is indispensable during spermatogenesis.

It has been reported that FA proteins regulate epigenetic programming during meiosis. Alavattam et al. found that FA core proteins (FANCA and FANCC) and FANCD2 regulated both H3K9me2 and H3K9me3, while FANCD2 regulated H3K4me2 independently of FA core complex [24]. Besides, FANCB was reported to regulate H3K9me2 and H3K9me3 on the sex chromosomes during meiosis [25]. Therefore, in the present study, we detected the epigenetic markers H3K9me2, H3K9me3, and H3K4me2 in *Fanci*^*−/−*^ spermatocytes. Despite the unaffected H3K9me2 in *Fanci*^*−/−*^ spermatocytes, our results clearly indicated that FANCI negatively regulated H3K9me3 and positively regulated H3K4me2 on XY chromatin in diplotene stage during meiosis. Notably, recent studies have indicated that the BRCA1-MDC1-RNF8 axis is upstream of the FA pathway in histone modification [40, 41]. Consistent with our results, FANCD2 was also reported to regulate RNF8-dependent histone modification H3K4me2, suggesting that RNF8 integrates the FA pathway and the BRCA1-MDC1-RNF8 axis [24]. Thus, our findings reinforce the intermediate role of RNF8 in this FA-DDR network, and further explorations are required to clarify the whole FA-DDR system during meiosis.

Despite the altered modification of H3K9me3 and H3K4me2 in diplotene, the meiotic progression was not arrested in *Fanci*^*−/−*^ mice. Besides, we observed the normal sex chromosome transcriptional inactivation in *Fanci*^*−/−*^ spermatocytes, indicating that the altered epigenetic modification did not result in transcriptional changes. It is possible that the varied epigenetic state in *Fanci*^*−/−*^ spermatocytes is associated with increased germ cell apoptosis.

Recently, it was reported that a *Fanci* knockout model in C57BL/6J mouse strain exhibited severe hypogonadism without any spermatocytes [29]. Our *Fanci*^*−/−*^ mice showed milder phenotypes by crossing C57BL/6J *Fanci*^*−/−*^ mice to ICR strains. Apart from germline defects, the mixed background *Fanci*^*−/−*^ mice in our study appeared to be healthy without any other known FA phenotypes. Notably, the difference between our study and the previous *Fanci* knockout model indicates the strain-dependent phenotypes. Previous studies have also revealed that FA gene knockout mice in mixed backgrounds exhibit much milder phenotypes than those in pure C57BL/6J strain [20, 21]. Our study also supports the point that strain background affects the phenotypic profiles in FA mouse models.

In summary, we have elucidated the important role and mechanism of FANCI in spermatogenesis. Our study provides new insights into the interaction between the FA pathway and meiotic epigenetic modification underlying spermatogenesis, which will reinforce the notion that DNA repair pathways play important roles in spermatogenesis and suggest the FANCI gene may be a candidate gene for human NOA.

## Supplementary information

Supplementary Figure Legends

S Fig 1

S Fig 2

S Fig 3

S Fig 4

S Fig 5
