# Correction: FANCI plays an essential role in spermatogenesis and regulates meiotic histone methylation

**DOI:** 10.1038/s41419-021-04096-7

**Published:** 2021-08-26

**Authors:** Lan Xu, Weiwei Xu, Duan Li, Xiaoxia Yu, Fei Gao, Yingying Qin, Yajuan Yang, Shidou Zhao

**Affiliations:** 1 Center for Reproductive Medicine, Cheeloo College of Medicine, Shandong University, Jinan, Shandong 250012 China; 2 Key laboratory of Reproductive Endocrinology of Ministry of Education, Shandong University, Jinan, Shandong 250012 China; 3 Shandong Key Laboratory of Reproductive Medicine, Jinan, Shandong 250012 China; 4 Shandong Provincial Clinical Research Center for Reproductive Health, Jinan, Shandong 250012 China; 5 National Research Center for Assisted Reproductive Technology and Reproductive Genetics, Shandong University, Jinan, Shandong 250012 China; 6 State Key Laboratory of Stem Cell and Reproductive Biology, Institute of Zoology, Chinese Academy of Sciences, Beijing, 100101 China; 7 University of Chinese Academy of Sciences, Beijing, 100101 China; 8 State Key Laboratory of Genetic Resources and Evolution, Kunming Institute of Zoology, Chinese Academy of Sciences, Kunming, Yunnan 650223 China; 9 Yunnan Key Laboratory of Animal Reproduction, Kunming Institute of Zoology, Chinese Academy of Sciences, Kunming, Yunnan 650223 China

**Keywords:** Cell death, Spermatogenesis

Correction to: *Cell Death and Disease* (2021) 12:780; 10.1038/s41419-021-04034-7, published online 9 August 2021

Since the publication of the article, the authors noticed that there are two affiliations missing for Dr. Yajuan Yang. The following affiliations need to be added: State Key Laboratory of Genetic Resources and Evolution, Kunming Institute of Zoology, Chinese Academy of Sciences, Kunming, Yunnan, 650223, China; and Yunnan Key Laboratory of Animal Reproduction, Kunming Institute of Zoology, Chinese Academy of Sciences, Kunming, Yunnan, 650223, China. The authors apologize for the mistake. The original article has been corrected.

