# Supplementary material for: FANCI plays an essential role in spermatogenesis and regulates meiotic histone methylation
Source: Cell Death Dis. 2021 Aug 9;12(8):780. doi: 10.1038/s41419-021-04034-7 (PMC8353022; doi:10.1038/s41419-021-04034-7)
Supplement: Supplementary file 1 — Supplementary Figure Legends [file 41419_2021_4034_MOESM1_ESM.docx]

**Supplementary Figure 1. Strategy for generating** ***Fanci-flag* knockin mice.** **a)** Specific DSB near the proposed insertion site were produced by CRISPR/Cas9 system, and the targeting vector containing 3×flag behind the start codon ATG was used for homologous recombination. The positions of primers are indicated by arrowheads. **b)** Sequence chromatograms of *Fanci-flag* knockin mice. The 3×flag sequence are indicated in black box. **c)** Genotyping of wild type mice, *Fanci-flag* heterozygous mice and *Fanci-flag* homozygous mice. **d)** Representative images of the wild-type and *Fanci-flag* mice. **e)** Representative images of the testes of wild-type and *Fanci-flag* mice. **f)** Body weights of wild-type and *Fanci-flag* mice at 8 weeks after birth. Six mice were analyzed for each group, and data are presented as mean ± SD. Student's t-test. **g)** Mean ratio of testis/body weight from 8 weeks old wild type and *Fanci-flag* mice. Six mice were analyzed for each group, and data are presented as mean ± SD. Student's t-test.

**Supplementary Figure 2. Strategy for generating *Fanci^-/-^* mice a)** The structure of mouse *Fanci* gene. The 98bp in exon 5 of *Fanci* gene was deleted in *Fanci^-/-^* mice by CRISPR/Cas9 technology. Blue box represents the deleted sequence. The positions of primers are indicated by arrowheads. **b)** The wild type FANCI protein and the predicted mutant protein in *Fanci^-/-^* mice. The deletion of 98bp causes a frame-shift mutation and results in a truncated protein of 116 amino acids (p.Pro103Cysfs*15). **c)** Genotyping of wild type mice, *Fanci^+/-^* mice and *Fanci^-^*^/-^ mice. **d)** Genotypes of offspring from heterozygous mating. A total of 308 mice were analyzed. **e)** Representative images of wild-type and *Fanci^-^*^/-^ mice. **f, g)** Representative images of the eye (f) and claw (g) of the wild-type and *Fanci^-^*^/-^ mice.

**Supplementary Figure 3. The expression of *Fanci* on purified germ cells during spermatogenesis. *Fanci* mean expression on all kinds of cells was used to normalize the results.**

**Supplementary Figure 4. Massive germ cell loss in 12 months old *Fanci^-/-^* mice. a)** H&E staining of testes and epididymides from 12 months old wild type and *Fanci^-/-^* mice. Scale bars, 50 μm. **b)** Quantification of different types of seminiferous tubules in 8 weeks old wild type and *Fanci^-/-^* mice. Six wild type mice and six *Fanci^-/-^* mice were analyzed. Data are presented as mean ± SD. *, *P* < 0.05. Chi-square test (Fisher’s exact test). **c)** Immunofluorescence staining for DDX4 (a germ cell marker) and SOX9 (a Sertoli cell marker) in wild type and *Fanci^-/-^* mice testes of 12 months old mice. Scale bars, 20 μm.

**Supplementary Figure 5. FANCI deletion increases apoptosis of germ cells. a)** Immunofluorescence staining against cleaved PARP1 (an apoptosis marker) on testes from 8-week old wild type and *Fanci^-/-^* mice. Scale bars, 50 μm. **b)** Frequencies of cleaved PARP1-positive tubules in wild type and *Fanci^-/-^* mice. In each group, six mice were analyzed. Data are presented as mean ± SD. *, Chi-square test (Fisher’s exact test). **c)** Numbers of cleaved PARP1-positive cells per c-PARP-positive tubule in wild type and *Fanci^-/-^* mice. Six wild type mice and six *Fanci^-/-^* mice were analyzed. Data are presented as mean ± SD. *, *P* < 0.05. Student's t-test. **d)** Immunofluorescence co-staining against cleaved PARP1 (an apoptosis marker) and PLZF (a marker for undifferentiated spermatogonia)/ SYCP3 (a marker for spermatocyte) /PNA (a marker for acrosome in R/E spermatids) on *Fanci^-/-^* testes. Scale bars, 50 μm. **e)** Proportion of cleaved PARP1-positive cells in *Fanci^-/-^* testes.
