# Supplementary figures and images for: FANCI plays an essential role in spermatogenesis and regulates meiotic histone methylation
Source: Cell Death Dis. 2021 Aug 9;12(8):780. doi: 10.1038/s41419-021-04034-7 (PMC8353022; doi:10.1038/s41419-021-04034-7)

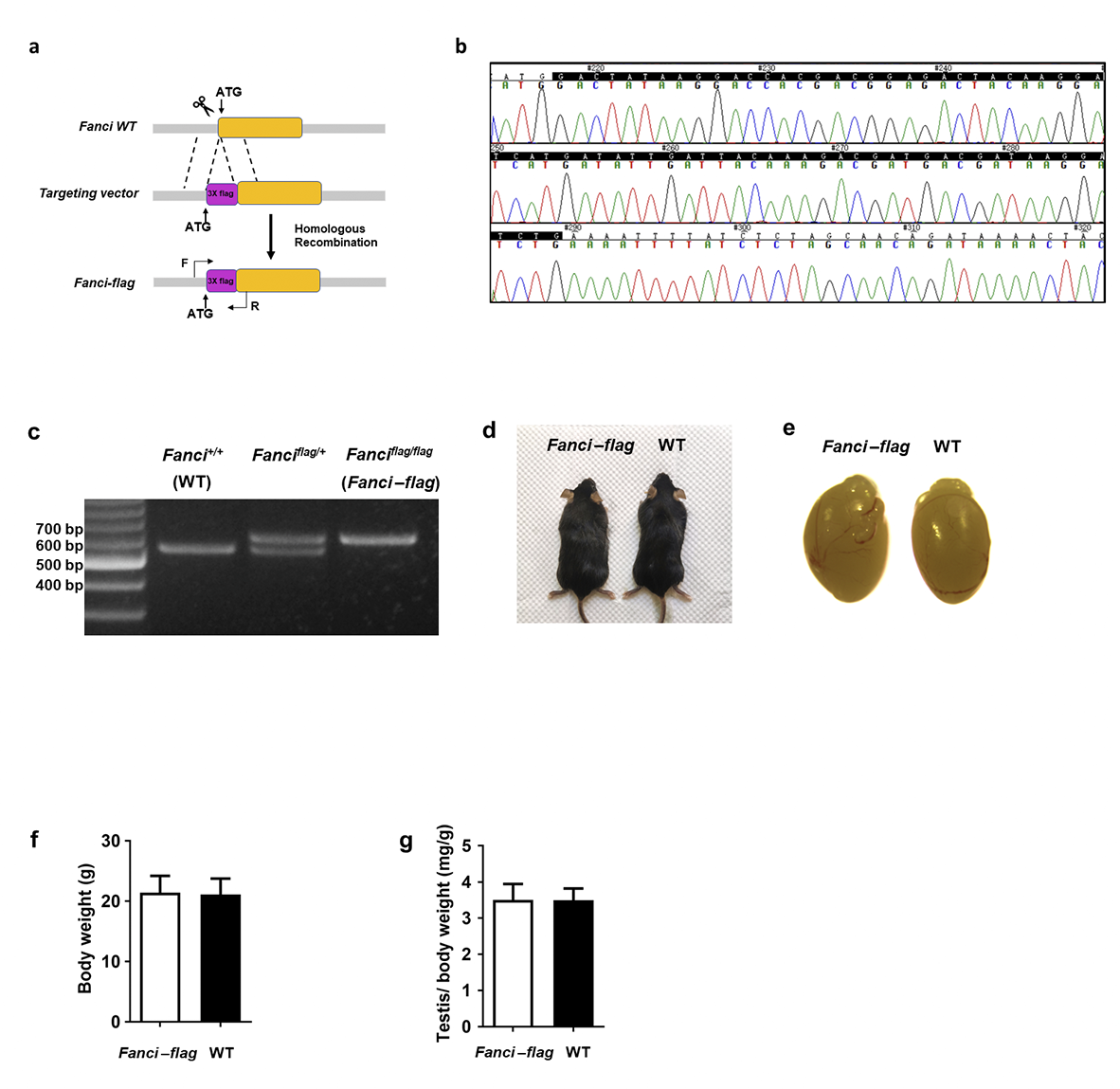

Supplement: Supplementary file 2 — S Fig 1 [file 41419_2021_4034_MOESM2_ESM.tif]

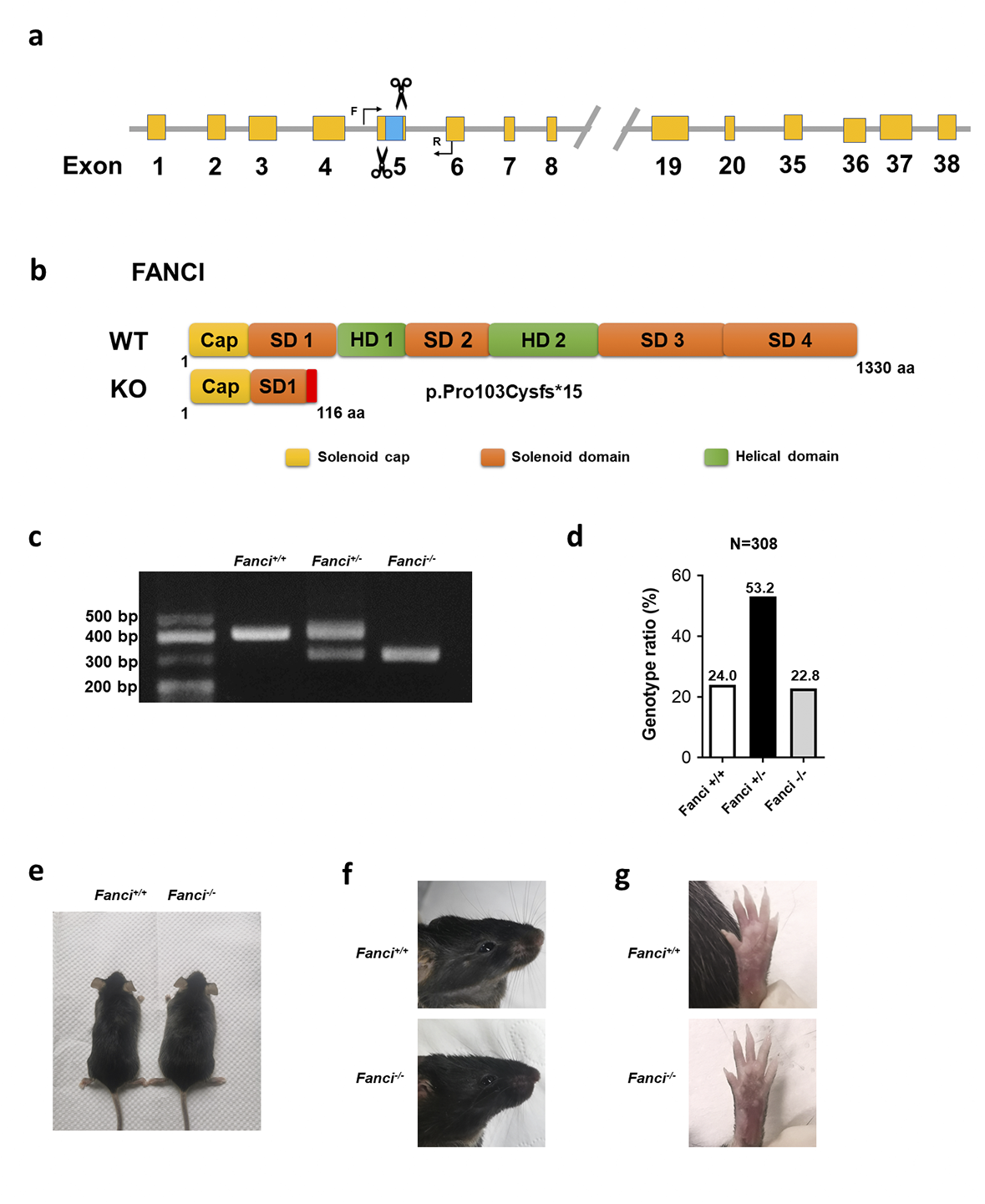

Supplement: Supplementary file 3 — S Fig 2 [file 41419_2021_4034_MOESM3_ESM.tif]

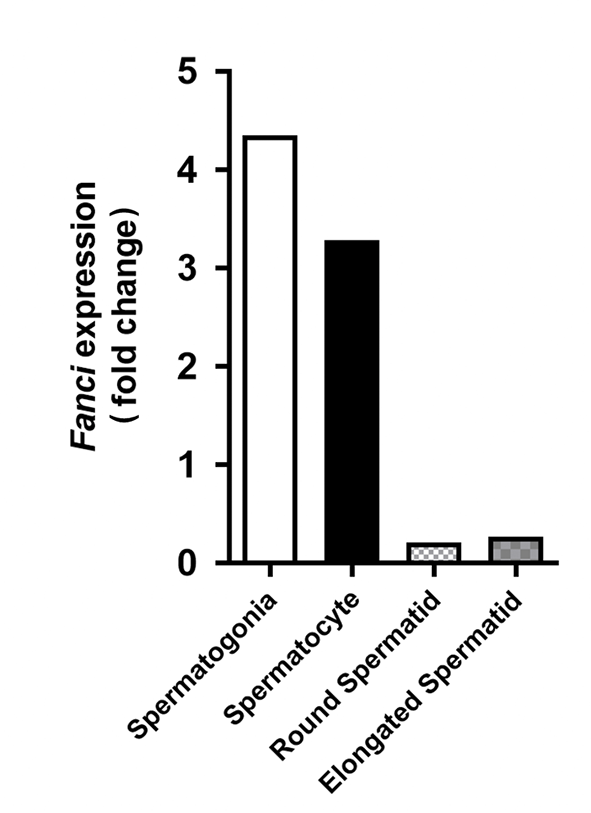

Supplement: Supplementary file 4 — S Fig 3 [file 41419_2021_4034_MOESM4_ESM.tif]

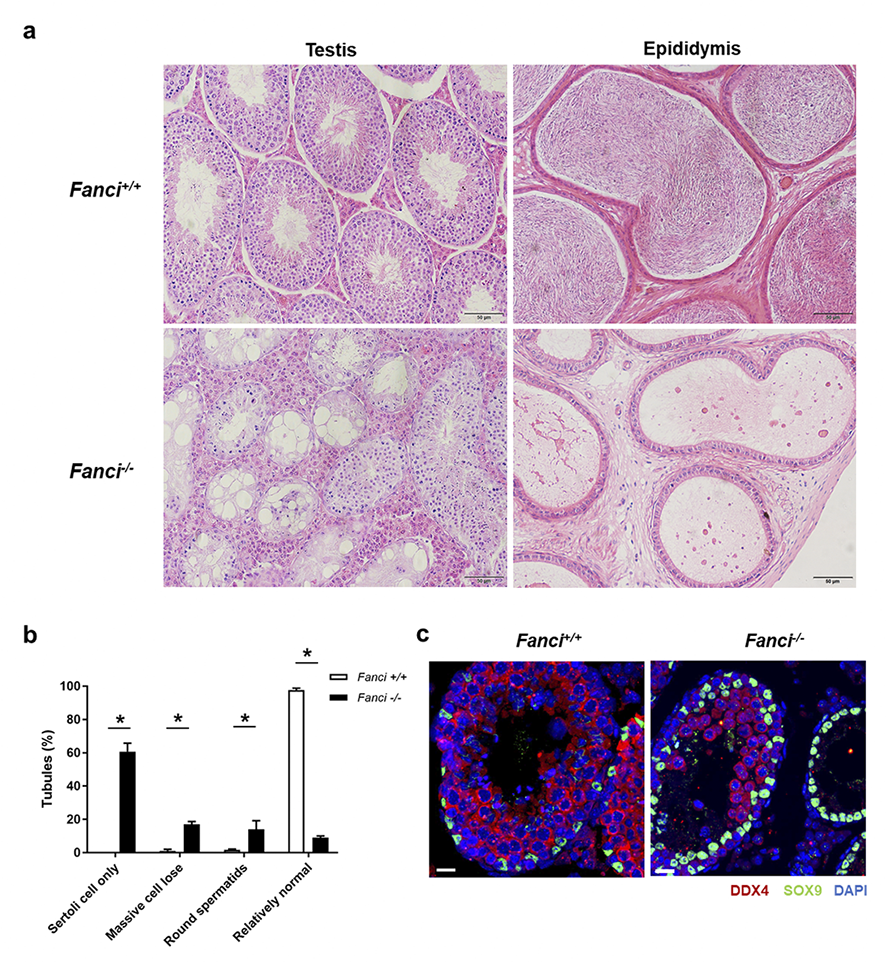

Supplement: Supplementary file 5 — S Fig 4 [file 41419_2021_4034_MOESM5_ESM.tif]

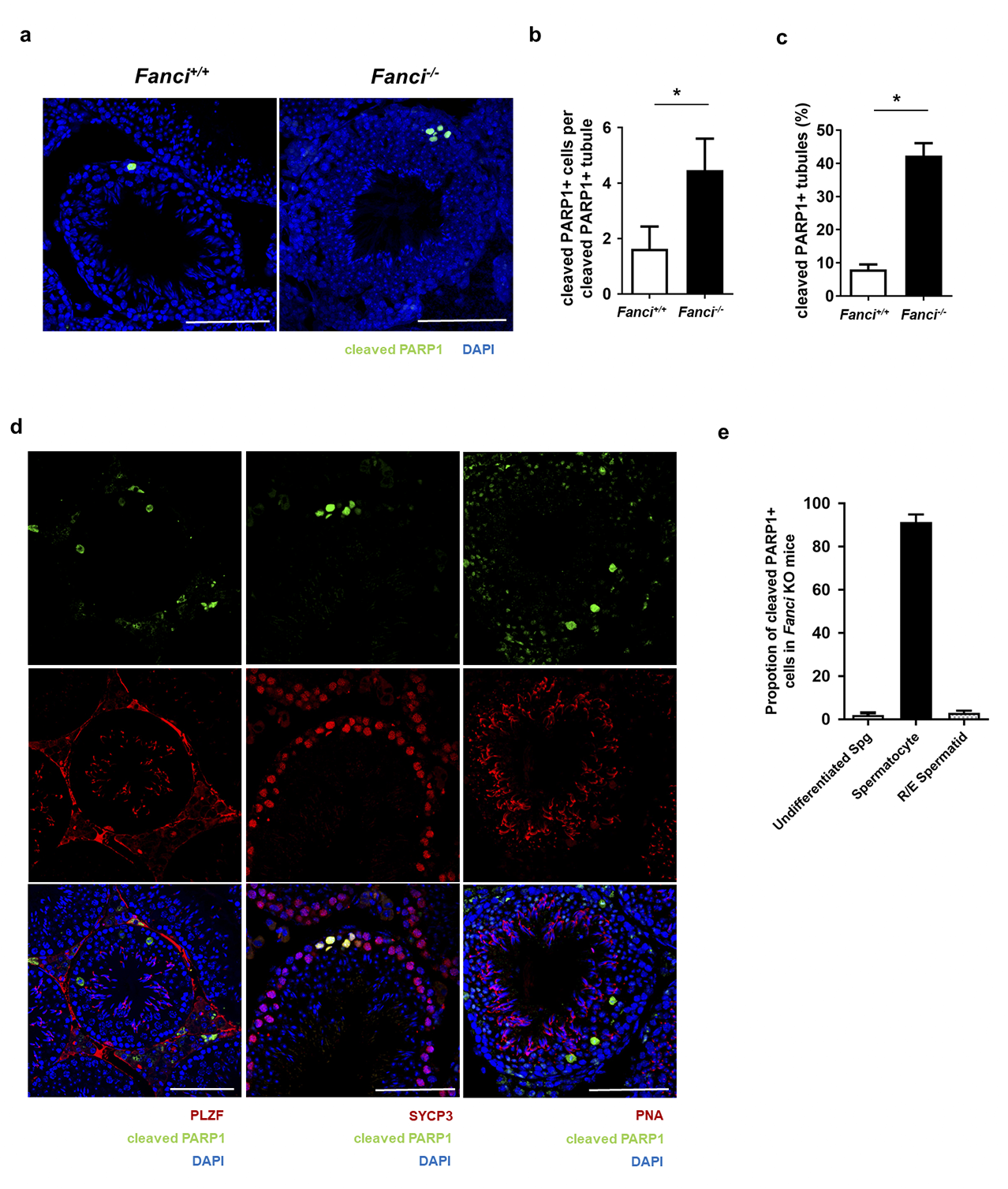

Supplement: Supplementary file 6 — S Fig 5 [file 41419_2021_4034_MOESM6_ESM.tif]
